# Risk factors for SARS-CoV-2 infection among health workers in India: a case control study

**DOI:** 10.3389/fpubh.2023.1156782

**Published:** 2023-05-31

**Authors:** Leyanna Susan George, Chitra Tomy, Charutha Retnakumar, Uday Narlawar, Pankaj Bhardwaj, Jayasree Krishnan, R. L. Lakshman Rao, Prakash Patel, Anil S. Bilimale, Poornima Baby, Minu Maria Mathew, Alessandro Cassini, Alice Simniceanu, Mo Yin, Benedetta Allegranzi, Mohammed Ahmad, Anisur Rahman, Syed Ahmed Mohiuddin, Sushama Thakre, Suman Suman Bhansali, Rajaat Vohra, Hari Krishnan, M. Logaraj, Vaishali Maheriya, Vaibhav Gharat, T. S. Dipu, Hilda Solomon, Sarita Sharma, M. Shwethashree, Rahul Hegde, Mohammad Waseem Faraz Ansari, Sanjeev Misra

**Affiliations:** ^1^Department of Community Medicine, Amrita School of Medicine, Amrita Vishwa Vidyapeetham University, Kochi, India; ^2^Scientist E, Indian Council of Medical Research, New Delhi, India; ^3^Department of Community Medicine, Government Medical College, Nagpur, India; ^4^Department of Community Medicine and Family Medicine, All India Institute of Medical Sciences Jodhpur, Jodhpur, India; ^5^Apollo Speciality Hospitals, Chennai, India; ^6^Osmania Medical College, Hyderabad, India; ^7^Community Medicine Department, Surat Municipal Institute of Medical Education and Research, Surat, India; ^8^School of Public Health and Department of Community Medicine, JSS Medical College, Mysuru, Karnataka, India; ^9^Department of Microbiology, Amrita School of Medicine, Amrita Vishwa Vidyapeetham University, Kochi, India; ^10^World Health Organization (Switzerland), Geneva, Switzerland; ^11^WHO Country Office, Delhi, India; ^12^Indira Gandhi Government Medical College and Hospital, Nagpur, India; ^13^SN Medical College, Jodhpur, India; ^14^Mahatma Gandhi Medical College and Hospital, Jaipur, India; ^15^SRM Institutes for Medical Science, SRM University, Chennai, India; ^16^GMERS Medical College, Sola, Ahmedabad, India; ^17^GMERS Medical College and Hospital Valsad, Valsad, India; ^18^Infectious Diseases, Amrita School of Medicine, Amrita Vishwa Vidyapeetham University, Kochi, India; ^19^Department of Community Medicine, KS Hegde Medical Academy, Nitte (Deemed to be University), Mangalore, India; ^20^ESIC Medical College, Gulbarga, India; ^21^All India Institute of Medical Sciences Jodhpur, Jodhpur, India

**Keywords:** COVID-19, health worker, nested case–control study, comorbidities, SARS-CoV-2

## Abstract

**Background:**

COVID-19 was declared as a Public Health Emergency of International Concern on 30th January 2020. Compared to the general population, healthcare workers and their families have been identified to be at a higher risk of getting infected with COVID-19. Therefore, it is crucial to understand the risk factors responsible for the transmission of SARS-CoV-2 infection among health workers in different hospital settings and to describe the range of clinical presentations of SARS-CoV-2 infection among them.

**Methodology:**

A nested case–control study was conducted among healthcare workers who were involved in the care of COVID-19 cases for assessing the risk factors associated with it. To get a holistic perspective, the study was conducted in 19 different hospitals from across 7 states (Kerala, Tamil Nadu, Andhra Pradesh, Karnataka, Maharashtra, Gujarat, and Rajasthan) of India covering the major government and private hospitals that were actively involved in COVID-19 patient care. The study participants who were not vaccinated were enrolled using the incidence density sampling technique from December 2020 to December 2021.

**Results:**

A total of 973 health workers consisting of 345 cases and 628 controls were recruited for the study. The mean age of the participants was observed to be 31.17 ± 8.5 years, with 56.3% of them being females. On multivariate analysis, the factors that were found to be significantly associated with SARS-CoV-2 were age of more than 31 years (adjusted odds ratio [aOR] 1.407 [95% CI 1.53–1.880]; *p* = 0.021), male gender (aOR 1.342 [95% CI 1.019–1.768]; *p* = 0.036), practical mode of IPC training on personal protective equipment (aOR 1. 1.935 [95% CI 1.148–3.260]; *p* = 0.013), direct exposure to COVID-19 patient (aOR 1.413 [95% CI 1.006–1.985]; *p* = 0.046), presence of diabetes mellitus (aOR 2.895 [95% CI 1.079–7.770]; *p* = 0.035) and those received prophylactic treatment for COVID-19 in the last 14 days (aOR 1.866 [95% CI 0.201–2.901]; *p* = 0.006).

**Conclusion:**

The study was able to highlight the need for having a separate hospital infection control department that implements IPC programs regularly. The study also emphasizes the need for developing policies that address the occupational hazards faced by health workers.

## Introduction

An outbreak of pneumonia of unknown etiology was initially reported in Wuhan city of China in December 2019 which was later confirmed to be a Novel coronavirus, known as severe acute respiratory syndrome coronavirus 2 (SARS-CoV-2) (1). Due to globalization, it spread fast across different parts of the world resulting in the World Health Organization (WHO) declaring COVID-19 as a Public Health Emergency of International Concern on 30th January 2020 (2).

SARS-CoV-2 is known to easily spread between people who are within a conversational distance (1 m) through short-range aerosol or droplet transmission. The virus is known to spread from one infected person to another while speaking, coughing, sneezing, or singing. The virus-containing aerosols or droplets when inhaled or coming in direct contact with the eyes, nose, or mouth can get a person infected. The aerosols can also remain suspended in the air or travel long distances (more than 1 m) in poorly ventilated or crowded indoor settings resulting in long-range aerosol transmission. It can also get transmitted when coming in contact with body fluids and contaminated surfaces. Aerosol-generating procedures in health facilities can also result in lead to long-range aerosol transmission (3, 4).

As on 30th January 2022, over 370 million confirmed cases and over 5.6 million deaths have been reported globally due to SARS-CoV-2 (5). Compared to the general population, healthcare workers and their families have been identified to be at a higher risk of getting infected with COVID-19. Even though healthcare workers represent less than 2–3% of the population, 14–35% of COVID-19 cases have been reported by WHO to be among health workers (6). Also, when compared to other occupational settings, COVID-19 outbreaks have been reported to be more common in the health sector. Other than, the presence of COVID-19 patients, the factors contributing to it are overcrowding, lack of space resulting in insufficient physical distancing with co-workers or patients, and lack of proper infection prevention control practices (IPC) (7). Inadequate availability of Personal Protective Equipment (PPE) was also found to contribute to the spreading of infections (8).

The role of hospital and healthcare workers is inevitable in flattening the epidemic curve of COVID-9. Since there was a drastic rise in the number of patients during the pandemic, the preparedness of the hospitals, proper IPC measures, and availability of sufficient healthcare workers for serving patients were all identified to be crucial factors for controlling the transmission of the infection. The pandemic created an extraordinary burden on an already fragile health system highlighting the need to improve the preparedness of the health system against it. Therefore, as a first step towards preparedness, it is important to understand the risk factors responsible for the transmission of SARS-CoV-2 infection among healthcare workers in different hospital settings and to describe the range of clinical presentation of SARS-CoV-2 infection among them. This will help in formulating appropriate guidelines and policy recommendations for the prevention of COVID-19 in hospital settings. Hence a multi-centric study across seven states of India was carried out to get an adequate representation of the healthcare workers who were actively involved in the management of COVID-19 in hospital settings.

## Methodology

A nested case–control study was conducted among healthcare workers who were involved in the care of COVID-19 cases for assessing the risk factors associated with it. To get a holistic perspective, the study was conducted among the major government and private hospitals that were actively involved in COVID-19 patient care. Hence, the health workers were recruited from 19 different hospitals across seven states (Kerala, Tamil Nadu, Andhra Pradesh, Karnataka, Maharashtra, Gujarat, and Rajasthan) of India. The study participants at each of the hospitals were enrolled using the incidence density sampling technique from December 2020 to December 2021. For a statistical power of 80%, a confidence interval of 95%, and an expected odds ratio of 2.5 for 63% exposed cases and 1 control per case the minimum sample size was calculated to be at least 50 cases and 50 controls at each selected site.

Even though this study was started before the rollout of COVID-19 vaccination, by early 2021 vaccination for health workers had begun, hence the study site investigators ensured that they only included health workers who were not vaccinated. Before the start of the study, the Medical Director/Superintendent and other administrative staff of each of the hospitals were briefed in detail about the study and its purpose. After obtaining ethical committee clearance from each of the hospitals and all necessary permissions, the study was initiated. In most of the selected hospitals, there was a separate fever clinic or designated area/ person to which each of the health workers, if suspected of contracting COVID-19, would report. The staff posted at these fever clinics were also briefed about the study and were asked to contact the site investigator as soon as a ‘case’ was reported. At some of the sites, the site investigators were regularly posted at these fever clinics and hence they were able to enroll the ‘cases’ as and when they reported. Additionally, a duty roster of health workers exposed to COVID-19 patients was also maintained by the infection control department of some of the hospitals to keep a close watch on the spread of infection. These rosters were also closely followed up by the site investigators to identify ‘cases’.

Once a health worker was identified as a case of COVID-19 in a healthcare setting (regardless of the type, location, and size of the healthcare facility/community) the research team approached the healthcare worker and invited them to participate in this study. “Incidence density sampling” was carried out for enrolling controls in parallel with cases. Control enrolment was determined by considering the facility as a whole attending to COVID-19 patients. Those health workers exposed to COVID-19 patients were identified based on the start and end dates of exposure according to the duty roster. All potential participants were screened for eligibility and controls were selected for each case.

Informed consent was obtained from both COVID-19-positive health workers and controls before recruitment to the study. All study participants were assigned a study identification number by the investigation team and that was used in all forms and samples to maintain anonymity. In order, to reduce bias, the interviewer was blinded to the classification of the interviewee as a case or control.

For this study, ‘health worker’ was defined as any member of staff in the health care facility involved in the provision of care for a COVID-19 patient, including those who have been present in the same area as the patient as well as those who may not have provided direct care to the patient but who have had contact with the patient’s body fluids, potentially contaminated items or environmental surfaces. This included health care professionals, allied health workers and auxiliary health workers such as cleaning and laundry personnel, x-ray physicians and technicians, clerks, phlebotomists, respiratory therapists, nutritionists, social workers, physical therapists, laboratory personnel, cleaners, admission/reception clerks, patient transporters, catering staff and so on. While ‘Exposure to COVID-19 patients’ was defined as Close contact (within 1 m and for more than 15 min) with a suspected/probable/confirmed COVID-19 patient(s) OR Indirect contact with fomites (for example, clothes, linen, utensils, furniture and so on) or with materials, devices or equipment linked to a suspected/probable/confirmed COVID-19 patient(s).

For enrolment, a ‘case’ was defined as a health worker who has been exposed in a healthcare setting to a COVID-19 patient, 14 days before the health worker testing positive either by RTPCR/RAT and being confirmed as a COVID-19 case. Those health workers having a confirmed COVID-19 case among their close contacts, including in their household, within the previous 14 days and those vaccinated who turned positive for COVID -19 were excluded from the analysis of risk factors and IPC measures and not from the general descriptive analysis.

While a ‘control’ was defined as a health worker exposed in a healthcare setting to a COVID-19 patient in the 14 days before recruitment and was never classified either as a suspect or probable or confirmed COVID-19 case. Controls were identified from the same settings/departments as from where the ‘cases’ were enrolled in order to ensure the same kind of chances of exposure as that of the cases. The controls were tested for seronegative using Wantai test kits. The cases and controls were enrolled in a 1:1 ratio and in some sites, 1:3 ratio of cases and controls were recruited.

At the time of enrolment, serum samples were collected and Form 1 was used to collect information from both cases and controls. Data regarding demographic information, symptom severity, medical history, use of medication, availability to IPC measures, adherence to IPC measures, and contact with and exposure to COVID-19 patients following their admission to the healthcare facility was collected. Detailed information regarding adherence to infection, prevention and control (IPC) measures was obtained and it consisted of the following: date of most recent IPC training, hours of cumulative IPC training on standard and additional precautions obtained, whether training was provided regarding the use of personal protective equipment (PPE), mode of training—remotely or in person and type of training-theory/practical/mix. The participant’s knowledge regarding hand hygiene practices and their routine use of alcohol-based hand rub/ soap in various instances such as before & after touching patient/patient’s surroundings, before cleaning/aseptic procedures, after exposure to body fluids etc. Additionally, whether they follow IPC standard precautions and the availability of alcohol-based hand rub at the point of care were also assessed. The pattern of use of PPE when indicated was assessed by asking whether they used it always based on risk assessment/most of the time/occasionally/rarely or never. PPE was defined as the use of any of the following based on risk assessment such as medical/surgical mask, face shield, gloves, goggles/glasses, gown, coverall, head cover, respirator (e.g., N95 or equivalent) and shoe covers were also assessed. They were asked whether sufficient quantity was available at the healthcare facility and if any of the above-mentioned PPE was ever missing. However, all this data was captured as reported by the study participants and was not cross-verified.

With regard to exposure to COVID-19 infected patients, information regarding the number of patients exposed too at the time of duty, close contact within 1 meter, number of times of exposure to an infected patient (< 10 times, 10–50 times, > 50 times), amount of time spent with the patient (< 5 min, 5–15 min, > 15 min), whether the participant had a prolonged face to face exposure of more than 15 min, and if yes whether they had used any PPE during the prolonged exposure and the type of PPE used was also ascertained. Details regarding the use of medical/surgical mask, gloves and respirator if test fitted at the time of exposure were also captured. Additionally, exposure to aerosolizing procedures and use of PPE at the time of exposure was assessed. The use of PPE in different scenarios such as exposure to patient’s materials/surroundings/materials soiled with the patient’s secretions etc. were also explored.

Follow-up data was collected 28 days later using Form 2 to collect information regarding their health status and symptom severity and a second serum sample was also collected. They were then transported to the microbiology lab where serum was separated and stored at 4°C or frozen to −20°C. The presence/absence of antibodies was confirmed by ELISA test using WANTAI SARS-CoV-2 Ab ELISA test kits.

The data retrieved was entered into an Excel sheet and analyzed using SPSS Version 20. Participants’ characteristics were analyzed in terms of mean and standard deviation, frequency, and percentages, respectively. The chi-square test or Fisher’s exact was done to find the association or mean difference for various risk factors for COVID-19 infection among cases and controls. The factors with a value of p less than 0.2 and those with no missing data were included in the multivariate logistic regression model. Multivariable logistic regression models with backward selection were used to determine the independent predictors for COVID-19 infection, expressed with odds ratios and their 95% confidence intervals.

## Results

A total of 973 healthcare workers were recruited for the study. Of which, 116, 125, 125, 211, 113, 196, 87 healthcare workers were each recruited from Kerala (case-50, control-66), Tamil Nadu (case-55, control-70), Telangana (case-50, control-75), Maharashtra (case-50, control-161), Karnataka (case-58, control-55), Rajasthan (case-50, control-146), Gujarat (case-32, control-55) respectively. By incident density sampling, a total of 345 healthcare workers suffering from COVID-19 were recruited to be compared with 628 incident-matched controls (1,1 ratio). The mean age of the participants was observed to be 31.17 ± 8.5 years, with 56.3% of them being females. When comparing the ages, it was observed that most of the participants were less than 31 years of age in both cases (57.7%) and control (68.3%; *p* = 0.001). While the number of males was higher in cases (49.3%) compared with controls (40.6%; *p* = 0.009). Most of the study participants were medical doctors (51.9%) by profession, followed by nurses (26.3%) and the majority of them had a tertiary/university level of education (93.3%). It was also observed that there was an equal distribution of healthcare workers among both groups based on their direct interaction with patients. However, these findings were found not to be significant. Details of these are provided in Table 1.

**Table 1:** Demographic characteristics of the enrolled health workers.

Variables	Cases (*N* = 345) *N* (%)	Controls (*N* = 628) *N* (%)	Chi-square value	*p* Value
*Age*				
≤ 31 years	199 (57.7)	429 (68.3)	10.997	**0.001**
> 31 years	146 (42.3)	199 (31.7)		
*Sex*				
Male	170 (49.3)	255(40.6)	6.804	**0.009**
Female	175 (50.7)	373(59.4)		
*Occupation*				
Doctors	335 (53.3%)	170 (49.0%)	2.03	0.730
Nurses/assistant nurse	163 (26.0%)	93 (27.0%)		
X-ray technician	16 (2.5%)	11 (3.2%)		
Lab personal	24 (3.8%)	17 (4.9%)		
Others^*^	90 (14.3%)	54 (15.7%)		
*Educational level*				
Primary	10 (2.9)	12 (1.9)		
Secondary	14 (4.1)	29 (4.6)	1.123	0.570
Tertiary/university	321 (93)	587 (93.5)		
*Direct interaction with patients*				
Direct interaction with patients	299 (86.7)	546 (86.9)		
			0.015	0.903
Indirect interaction with patients	46 (13.3)	82 (13.1)		

* Physical therapist, Nutritionist/dieticians, Admission/reception clerk, Patient transporter, Catering staff, Cleaner, Administration/clerk, Security.

When comparing the Infection, prevention and control (IPC) practices and training received by the health workers, it was observed that the majority of cases (84%) and controls (84.87%) have received recent IPC training within the healthcare facility (*p* = 0.736) but only 66.1% of cases and 66.6% of controls followed the IPC standard precautions when in contact with a patient always as recommended (*p* = 0.313). A total of 150 (43.5%) cases and 230 (36.7%) controls had less than 2 h of IPC training received within the health care facility (*p* = 0.059). However, it was observed that about half of the cases (49.3%) and controls (57.5%) had received both theoretical and practical IPC training on personal protective equipment (*p* = 0.05). The majority of cases (97.68%) and controls (97.13%) had knowledge of the steps of hand hygiene practices (*p* = 0.612); however, only 68.9% of cases and 67.19% of controls were following all the recommended hand hygiene practices (*p* = 0.861). The proportion of the cases and controls using alcohol-based hand rub/ soap and water always before touching the patient (case-67.2%, control-68.8%), after touching the patient (case-74.2%, control-76.6%), before cleaning/aseptic procedures (case-74.8%, control-74.5%), after (risk of) body fluid exposure (case-79.4%, control-81.4%), after touching a patient’s surroundings (case-67.2%, control-67.7%), showed no significant difference between cases and controls. Almost all the cases and controls reported that PPE (case-91.6%, control-91.7%) and alcohol-based hand rubs (case-96.8%, control-95.2%) were available at point of care in the health care facilities; however, only 66.1% of cases and 75.5% of controls were wearing PPE always according to risk assessment (*p* = 0.603). Details of these are provided in Table 2.

**Table 2:** Distribution of study participants based on Infection, prevention and control (IPC) measures.

Variables	Case (*N* = 345) *N* (%)	Controls (*N* = 628) *N* (%)	Chi-square value	*p* Value
*Received recent IPC training within the healthcare facility*				
Yes	290 (84)	533 (84.87)	0.113	0.736
No	55 (16)	95 (15.13)		
*Hours of IPC training received within the healthcare facility*				
Less than 2 h	150 (43.5)	230 (36.7)	5.651	0.059
More than 2 h	140 (40.6)	303(48.2)		
I do not know what IPC standard precautions are	55 (15.9)	95 (15.1)		
*Type of IPC training on personal protective equipment*				
Only remotely/theoretical	85 (24.6)	132(21)		
Only practical	34 (9.9)	40(6.4)		
Both	170 (49.3)	361(57.5)	7.789	**0.05**
I do not know what IPC standard precautions are	56 (16.2)	95(15.1)		
*Knowledge of moments of hand hygiene in healthcare*				
Yes	337(97.68)	610(97.13)	0.257	0.612
No	8(2.32)	18(2.87)		
*Follow recommended hand hygiene practices*				
Always, as recommended	238 (68.9)	422 (67.19)	0.751	0.861
Most of the time	94 (27.3)	177 (28.18)		
Occasionally	11 (3.2)	26 (4.14)		
Rarely	2 (0.6)	3 (0.4)		
*Follows IPC standard precautions when in contact with any patient*				
Always, as recommended	228 (66.1)	418(66.6)		
Most of the time	81 (23.5)	149(23.7)		
Occasionally	8 (2.3)	24(3.8)		
Rarely	0	3 (0.5)		
Never	7 (2)	7 (1.1)	5.927	0.313
I do not know what IPC standard precautions are	21 (6.1)	27(4.3)		
*Wearing PPE as indicated*				
Always, according to the risk assessment	228 (66.1)	474(75.5)	2.737	0.603
Most of the time, according to the risk assessment	81 (23.5)	108(17.2)		
Occasionally	8 (2.3)	20(3.2)		
Rarely	7 (2)	7(1.1)		
Never	21 (6.1)	19(3)		

Table 3 describes the IPC measures taken by the study participants on exposure to COVID-19 patients. The table describes the IPC and PPE measures taken during close contact with the patient (within 1 m), during direct contact with the patient’s materials and during contact with the surfaces around the patient. It was observed that 48.98% of cases and 49.2% of controls were specifically dedicated to COVID-19 patient care with a mean duration of 9 days (± 4.8). However, more than half of the cases (60.86%) and controls (68%) had received specific training in COVID-19 patient care (*p* = 0.025). Even though, three fourth of the cases (79.1%) and controls (75.3%) stated a history of exposure to COVID-19 patient care (*p* = 0.179), only a quarter of the cases (23.76%) and controls were exposed to COVID-19 cases (25.14%) outside of duties (*p* = 0.630). It was also observed, that a significant (*p* = 0.006) number of cases (42.6%) and controls (37.8%) avoided using public transport in order to prevent getting infected, even though there was no significant change in their social interactions in the last 14 days (*p* = 0.453). History of exposure to COVID-19 patient was classified into the history of close contact with the patient (within 1 m), direct contact with the patient’s materials, and contact with the surfaces around the patient. About 239 cases (69.2%) and 405 controls (64.5%) had a *history of close contact with the patient* (*p* = 0.131). Among those with a history of close contact with the patient, no significant difference was observed between cases and controls with regard to the number of times of close contact with the patient, the maximum amount of time spent with a COVID-19 patient, history of prolonged face-to-face exposure (> 15 min), the performance of hand hygiene before and after contact with the patient, an aerosolizing procedure performed on the patient and history of contact with the patient’s body fluids. About 161 cases (46.7%) and 261 controls (41.6%) had a history of direct contact with the patient’s materials (*p* = 0.124). Among those with a history of direct contact with the patient’s materials, no significant difference was observed between cases and controls with regard to the number of times of exposure to the patient’s materials, history of contact with the patient’s body fluids through the patient’s materials, and performance of hand hygiene before and after contact with the patient’s materials. About 167 cases (48.5%) and 265 controls (42.19%) had a *history of direct contact with the surfaces around the patient* (*p* = 0.062). Among those with a history of direct contact with the surfaces around the patient, no significant difference was observed between cases and controls about the number of times of direct contact with the surfaces around the patient, history of contact with the patient’s body fluids through the surfaces around the patient and performance of hand hygiene before and after contact with these surfaces. No significant difference was observed between cases and controls with regard to PPE usage during prolonged face-to-face exposure to COVID-19 patients/ when in contact with the patient’s body fluids *via* the patient’s materials/ contact with the patient’s body fluids *via* the surfaces around the patient. Similarly, the removal of gloves after contact with the patient and the type of materials used for hand hygiene before and after contact with the patient/ patient’s materials/ surfaces around the patient also had no significant difference between cases and controls.

**Table 3:** Infection Prevention Control (IPC) measures taken by study participants on exposure to COVID-19 infected patient(s).

Characteristics	Case (*N* = 345) *N* (%)	Controls (*N* = 628) *N* (%)	Total(*N* = 973) *N* (%)	Chi-square value	*p* Value
Received Specific training in COVID-19 patient care					
Yes	210 (60.86)	427 (68)	637 (65.5)	4.999	**0.025**
No	135 (39.14)	201 (32)	336 (34.5)		
Use of Public transport in the last 14 days					
Most days (≥ 8 days)	36 (10.4)	71 (11.3)	107 (10.9)		
Some days (4–7 days)	42 (12.2)	92(14.6)	134 (13.8)		
Few days (≤ 3 days)	120 (34.8)	228 (36.3)	348 (35.8)		
Not used public transport	147 (42.6)	237 (37.8)	384 (39.5)	12.532	**0.006**
Exposure to COVID-19 patient					
Yes	273 (79.1)	473(75.3)	746 (76.7)		
No	72 (20.9)	155(24.7)	227 (23.3)	1.809	0.179
*H/o close contact (within 1 m) with the patient(s) since their admission*					
H/o close contact (within 1 m) with the patient(s) since their admission					
Yes No	239 (69.2) 106 (30.8)	405(64.5) 223(31.7)	644 (66.2) 329 (33.8)	2.278	0.131
No. of times of close contact with patient (total; case, *n* = 239, control, *n* = 405)					
< 10 times 10–50 times > 50 times	96 (40.2) 87 (36.4) 56 (23.4)	145(35.8) 163(40.2) 97(24)	241 (37.4) 250 (38.8) 153 (23.8)	1.355	0.508
Maximum amount of time spent with a COVID-19 patient (case, *n* = 239, control, *n* = 405)					
< 5 min 5–15 min > 15 min	61 (25.5) 80 (33.5) 98 (41)	101(24.9) 164(40.5) 140(34.6)	162 (25.2) 244 (37.9) 238 (36.9)	3.661	0.160
H/o prolonged face-to-face exposure (> 15 min) (case, *n* = 239, control, *n* = 405)					
Yes No	129 (53.9) 110 (46.1)	233(57.5) 172 (42.8)	362 (56.2) 282 (43.8)	0.772	0.380
PPE^*^ usage when in prolonged face to face exposure to Covid 19 patients (case, *n* = 129, control, *n* = 233)					
Yes No	125 (96.9) 4 (1.2)	223(35.5) 10 (1.6)	348 (96.1) 14 (3.9)	0.317	0.574
Performance of hand hygiene^**^ before contact with the patient (case, *n* = 239, control, *n* = 405)					
Always, as recommended	169 (70.7)	287(45.7)	456 (70.8)		
Most of the time	43 (18)	89(22)	132 (13.6)		
Occasionally	11 (4.6)	9(2.2)	20 (2.1)		
Rarely	5 (2.1)	9(2.2)	14 (1.4)		
Never	11 (4.6)	11(2.7)	22 (2.3)	5.484	0.241
Performance of hand hygiene^*^ after contact with the patient (case, n = 239, control, n = 405)					
Always, as recommended	194 (81.2)	339(83.7)	533 (82.8)		
Most of the time	38 (15.9)	57(14.1)	95 (14.8)		
Occasionally	2 (0.8)	6(1.5)	8 (1.2)		
Rarely	0	1(0.2)	1 (0.1)		
Never	5 (2.1)	2(0.3)	7 (0.7)	5.081	0.279
Aerosol procedure performed on the patient (case, *n* = 239, control, *n* = 405)					
Yes No	83 (34.7) 156 (65.28)	146(36) 259 (63.96)	229 (35.6) 415 (64.4)	0.115	0.735
Wear PPE while performing aerosol procedure (case, *n* = 239, control, *n* = 405)					
Yes No	82 (98.8) 1 (0.3)	135(92.5) 11(7.6)	217 (94.8) 12 (3.9)	3.090	0.079
H/O contact with the patient’s body fluids (case, *n* = 239, control, *n* = 405)					
Yes No	109(45.6) 130(54.4)	158(39) 247(61)	267 (41.5) 377 (58.5)	2.693	0.101
Wearing PPE when in contact with patients’ body fluids (case, *n* = 109, control, *n* = 158)					
Yes	104 (95.4)	153(96.8)	257 (96.3)	0.075	0.784
No	5(4.6)	5(3.2)	10 (3.7)		
H/O direct contact with the patient’s materials					
H/O direct contact with the patient’s materials					
Yes	161(46.7)	261(41.6)	422 (43.45)		
No	180(52.2)	342(54.5)	522 (53.6)	7.503	**0.023**
Unknown	4(1.2)	25(4)	29 (3)		
Number of times of exposure to patients materials (case, *n* = 161, control, *n* = 261)					
< 10 times 10–50 times > 50 times	76(47.2) 56(34.8) 29(18)	119(45.6) 113(43.3) 29(11.1)	195 (46.2) 169 (40) 58 (13.7)	5.308	0.070
H/O contact with the patient’s body fluids *via* the patient’s materials (case, *n* = 161, control, *n* = 261)					
Yes No	55(34.2) 106(65.9)	83(31.8) 178 (68.2)	138 (32.7) 284 (67.3)	0.252	0.616
Wearing PPE^*^ when in contact with the patient’s body fluids *via* the patient’s materials (case, *n* = 55, control, *n* = 83)					
Yes No	53(96.4) 2(3.6)	80(96.4) 3(3.6)	133 (96.4) 5(3.6)	0.000	0.995
Performance of hand hygiene^**^ before coming into contact with the patient’s materials (case, *n* = 161, control, *n* = 261)					
Always, as recommended	104(64.5)	159(60.9)	263 (62.3)		
Most of the time	35(21.6)	65(24.9)	100 (23.7)	0.669	0.955
Occasionally	8(4.9)	14(5.4)	22 (5.2)		
Rarely	3(2.3)	5(1.9)	8 (1.9)		
Never	11(6.7)	18(6.9)	29 (6.9)		
Performance of hand hygiene^**^ after contact with the patient’s materials (case, *n* = 161, control, *n* = 261)					
Always, as recommended Most of the time Occasionally Rarely Never	116 (72.04) 37(22.9) 6(3.7) 1(0.6) 1(0.6)	199(76.2) 49(18.7) 7(2.7) 1(0.4) 5(1.9)	315 (74.6) 86 (20.4) 13 (3.1) 2 (0.5) 6(1.4)	2.745	0.601
H/O direct contact with the surfaces around the patient					
H/O direct contact with the surfaces around the patient					
Yes No	167(48.4) 178(51.5)	265(42.2) 363(57.8)	432 (44.4) 541 (55.6)	3.477	0.062
Number of times of direct contact with the surfaces around the patient (case, *n* = 167, control, *n* = 265)					
< 10 times 10–50 times > 50 times	84 (50.3) 59(35.3) 24(14.4)	122(46) 110(41.5) 33(12.5)	206 (47.7) 169 (39.1) 57 (13.2)	1.676	0.433
H/O contact with the patient’s body fluids *via* the surfaces around the patient (case, *n* = 167, control, *n* = 265)					
Yes No	39(23.2) 128(76.7)	62(23.4) 203(76.6)	101 (23.4) 331 (76.6)	0.0001	0.992
Wearing PPE^*^ while in contact with the patient’s body fluids *via* the surfaces around the patient (case, *n* = 39, control, *n* = 62)					
Yes No	39(100) 0	58(93.5) 4(6.5)	97 (96) 4 (3.9)	1.198	0.274
Performance of hand hygiene^**^ after contact with these surfaces (case, *n* = 167, control, *n* = 265)					
Always, as recommended Most of the time Occasionally Rarely Never	120(71.9) 33(19.8) 6(3.6) 2(1.2) 6(3.6)	189(71.3) 47(17.7) 12(4.5) 0 17(6.4)	309 (71.5) 80 (18.5) 18 (4.2) 2 (0.5) 23(5.3)	5.152	0.272

* Personal protective equipment (PPE) included Medical/surgical mask, Face shield, gloves, goggles/glasses, gown, headcover, Respirator, shoe cover.

** Hand hygiene material included alcohol-based hand rub, soap and water.

Among cases, 206 (59.7%) and among controls, 321 (51.1%) had a history of co-morbidities (*p* = 0.011). Diabetes mellitus was more among cases (3.47%) than controls (1.2%) while Obesity was more among controls (40.8%) than in cases (33.2%). However, both Diabetes (*p* = 0.015) and obesity (*p* = 0.033) were found to be statistically significant. Even though the proportion of healthcare workers with asthma and hypertension was more among cases than in controls they were not statistically significant (Table 4).

**Table 4:** Distribution of study participants based on co-morbidities.

Co-morbidity	Case (*N* = 345) *N* (%)	Controls (*N* = 628) *N* (%)	Chi-square	*p* Value
Presence of Co-morbidities				
Yes	206 (59.7)	321 (51.1)	6.627	**0.011**
Obesity				
Obese Normal	96 (33.2) 193(66.8)	207 (40.8) 300(59.2)	4.522	**0.033**
Diabetes				
Yes	12 (3.47)	7 (1.2)	6.497	**0.015**
Heart disease				
Yes	2 (0.57)	5 (0.79)	0.146	0.702
Asthma (requiring medication)				
Yes	8 (2.3)	12 (1.9)	184	0.668
Hypertension				
Yes	10 (2.89)	12 (1.91)	0.983	0.321
Chronic kidney disease				
Yes	2 (0.58)	1 (0.16)	1.281	0.288
Hypothyroidism				
Yes	11 (3.2)	16 (2.5)	0.339	0.561

**Figure 1:**
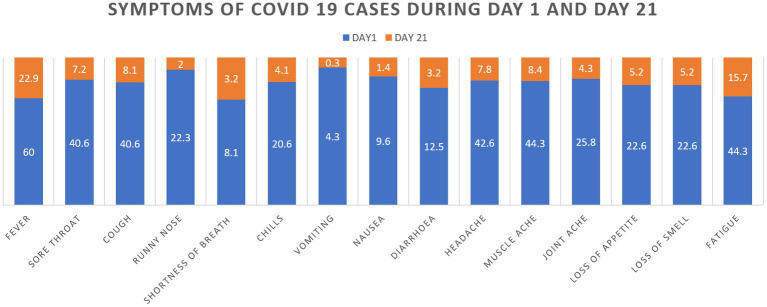
Distribution of cases based on symptoms on day 1 and day 21.

Among the 345 COVID-19 cases, 306 (88.7%) of them had reported having clinical symptoms on the first day of which only 117 (33.9%) had persistent symptoms at day 21. The common clinical symptoms experienced by the COVID-19 healthcare workers during day 1 and follow-up on day 21 were, fever (60 vs. 22.9%), Sore throat (40.6 vs. 7.2%), Cough (40.6 vs. 8.1%), Runny nose (23.2 vs. 2%), Chills (20.6 vs. 4.1%), Headache (42.6 vs. 7.8%), Muscle ache (44.3 vs. 8.4%), Joint ache (25.8 vs. 4.3%), Loss of appetite (22.6 vs. 5.2%), Loss of smell (anosmia) or taste (22.6 vs. 5.2%) and fatigue (44.3 vs. 15.7%; Figure 1).

About 46 cases (13.3%) and 51 (8.1%) controls had not taken any medication apart from those for COVID-19 (*p* = 0.009). It was observed that the use of prophylactic treatment was quite high among the cases than the controls (13.9 vs. 7.48%, *p* = 0.001). The most commonly used prophylactic medicines were ayurvedic and homeopathic medicines and among modern medicines, it was observed that chloroquine was quite prevalent.

On multivariate analysis of factors associated with SARS-CoV-2 infection among health care workers, there was a significant association between age (adjusted odds ratio [aOR] 1.407 [95% CI 1.53–1.880]; *p* = 0.021), male gender (aOR 1.342 [95% CI 1.019–1.768]; *p* = 0.036), practical mode of IPC training on personal protective equipment (aOR 1.935 [95% CI 1.148–3.260]; *p* = 0.013), exposure to COVID-19 patient (aOR 1.413 [95% CI 1.006–1.985]; *p* = 0.046), presence of diabetes mellitus (aOR 2.895 [95% CI 1.079–7.770]; *p* = 0.035) and those received prophylactic treatment for COVID-19 in the last 14 days (aOR 1.866 [95% CI 1.201–2.901]; *p* = 0.006). On the contrary, used public transport on most days (≥ 8 days; aOR 0.483 [95% CI 0.287–0.812]; *p* = 0.006), few days (≤ 3 days; aOR 0.630 [95% CI 0.418–0.948]; *p* = 0.027) reduced the risk of SARS-CoV-2 infection among health care workers (Table 5).

**Table 5:** Univariate and multivariable logistic regression to find the independent risk factors for SARS-COV 2 infection among healthcare workers in India.

Characteristics	Crude odds ratio	*p* Value	Adjusted odds ratio	*p* Value
Age				
> 31 years ≤ 31 years	1.582 (1.205–2.076) 1	0.001	1.407(1.53–1.880) 1	0.021
Sex				
Male Female	1.421(1.091–1.851) 1	0.009	1.342(1.019–1.768) 1	0.036
Use of public transport in the last 14 days				
Most days (≥ 8 days) Some days (4–7 days) Few days (≤ 3 days) Not used public transport	0.523(0.321–0.851) 0.654(0.351–1.217) 0.606(0.406–0.904) 1	0.009 0.180 0.014	0.483(0.287–0.812) 0.662(0.348–1.258) 0.630(0.418–0.948) 1	0.006 0.208 0.027
IPC training method on personal protective equipment				
Only remotely/theoretical Only practical Do not know about IPC standard precautions are Both theoretical and practical	1.367(0.985–1.898) 1.805(1.103–2.953) 1.252 (0.858–1.825) 1	0.061 0.019 0.243	1.373(0.979–1.925) 1.935(1.148–3.260) 1.205(0.805–1.802) 1	0.066 0.013 0.365
Exposure to COVID-19 patient				
Yes No	1.243(0.905–1.706) 1	0.179	1.413(1.006–1.985) 1	0.046
Diabetes				
Yes No	3.197(1.247–8.197) 1	0.016	2.895(1.079–7.770) 1	0.035
Received Prophylactic treatment for COVID-19 in the last 14 days				
Yes No	1.998(1.305–3.058) 1	0.001	1.866(1.201–2.901) 1	0.006

## Discussion

This multicenter study was one of the largest case–control study conducted among health workers in India to assess the risk for the transmission of COVID-19. It was conducted among a total of 973 health workers from 19 different hospitals across 7 states of India. It was observed that health workers who were males, above the age of 31 years, and had exposure to COVID-19 patients were found to be at a higher risk of contracting COVID-19 infection. However, health workers who had received some form of IPC training and those who had taken prophylactic treatment for COVID-19 were found to be infected more. This could probably be due to inadequate IPC training and complacency developed as a result of feeling protected by taking prophylactic treatment.

When comparing the odds of developing COVID-19 among the different sexes, males were found to be more susceptible. This could probably be a result of the biological differences in the immune system’s ability to fight against the SARS-2-CoV-2 infection (9, 10). Literature has shown that men were found to be more susceptible as a result of a multitude of factors such as sex hormones, higher expression of coronavirus receptors (ACE 2) in men (9–13) and as a result of lifestyle factors. When compared to females, men tend to have higher levels of smoking and drinking making them more vulnerable to infections. Additionally, women tend to be more responsible in following the COVID-19 protocols such as frequent hand washing, wearing of face masks, and stay-at-home orders thereby resulting in men being at a higher risk than women (9, 14, 15). Griffith et al. (16) too stated that males were more likely to be infected as a result of genetics, psychosocial, and behavioral characteristics. Increase in age was also identified as a significant factor in this study. Participants over the age of 31 were more likely to contract COVID-19 and several studies also have pointed out that people became more susceptible to COVID-19 infections and their long-term implications as they got older (17–19).

Previous studies had shown that the use of public transport increases the risk of contracting COVID-19 infection (20–22). However, in this study it was observed that the majority of the study participants who were infected with COVID-19 had not used any public transport indicating that they may have been infected from the hospital setting. Hence, this study captured the risk of COVID transmission occurring within the hospitals thereby reflecting upon the prevailing IPC practices in these settings.

Training programs on ‘Infection, prevention and control’ practices have been found to minimize the risk of transmission of infection among health workers. Especially when it is designed to emphasize the risks and implications of poor IPC practices (23). It was observed that the risk of infection was low among those health workers who had received both theory and practical training sessions on IPC. Those who had received only practical demonstration of personal protective equipment without theoretical knowledge were found to have a significantly higher chance of developing SARS-CoV2 infection. Thereby highlighting that hands-on training when given along with theoretical orientation was found to be more effective. Those with prior knowledge and a positive attitude towards IPC practices were found to follow correct IPC practices when compared to those with no prior exposure to IPC practices. This was particularly evident in the correct use of PPE and carrying out WASH techniques (water, sanitation and hygiene). It is observed that those with no prior training or exposure were found to not follow the correct IPC practices (24). The findings of the study were in line with a systematic literature review where it clearly stated that creating awareness regarding IPC and highlighting the dangers of inadequate IPC practices were both found to be potential factors that influenced IPC compliance (25).

Even though the majority of healthcare workers had undergone recent IPC training within the healthcare facility, it was reported that most of them were not following these practices when coming in contact with patients. This emphasizes the need for repeated training for bringing about behavioral change among them. Additionally, supportive supervision and monitoring of healthcare workers to ensure IPC compliance would provide better outcomes. It was observed that the majority of cases and controls had adequate knowledge of the steps of hand hygiene practices; however, only two-thirds of them were following the recommended hand hygiene practices. This suggests that having knowledge is not enough, instead, it requires motivation and behavioral change to incorporate these practices into their daily work practices (26). Almost all the cases and controls reported that PPE at the point of care was available in the health care facilities; however, only 66.1% of cases and 75.5% of controls were wearing PPE according to risk assessment. According to the National Institute for Occupational Safety and Health (NIOSH) Hierarchy of controls, PPE was proven to be least effective than other measures in the IPC hierarchy of measures to protect healthcare workers from occupational hazards (27). Hence, WHO has also suggested that in addition to IPC practices and the use of PPE, hospitals should encourage the incorporation of appropriate administrative, engineering, and environmental control measures (28).

This study was able to identify exposure to COVID-19 patients as an occupational risk factor for the health workers exposed to them and this finding has been supported by Lenggenhager et al. (29) in his study where exposure to COVID-19 was found to be a significant risk factor Additionally, this study was able to identify comorbidities such as Diabetes to significantly increase the risk of contracting SARS-CoV2 infection. Diabetes has been shown to affect viral entrance into cells and the inflammatory response to infection in experiments (30). Other reviews and meta-analyses found that hypertension and diabetes were the most common comorbidities associated with SARS-CoV2 infection, followed by cardiovascular disease. SARS-CoV2 infection has also been linked to respiratory illness (31). Comorbidities such as asthma and hypertension were more common in cases than in controls in our study, although the differences were not statistically significant. This could be because there were fewer study participants with various comorbidities in our study.

Obesity is another risk factor that is found to be fast growing in the pandemic situation and it has been found to be a major risk factor for the development of COVID-19 infection. Studies have shown that obesity weakens the immune system, which makes individuals susceptible to SARS COV 2 infection (32–35). However, in our study obesity was not identified as a significant risk factor for SARS-CoV2 infection.

It was observed that the use of prophylactic treatment was quite high among the cases than the controls. The study also observed that participants who had received prophylactic treatments for COVID-19 in the last 14 days were found to have a higher risk of getting affected by COVID-19. The carelessness and non-adherence to IPC practice as a result of complacency due to prophylactic treatment may have increased COVID-19 infections. Even though hydroxychloroquine was advocated in the early stages of the COVID-19 pandemic (36), it was later withdrawn based on new evidence that emerged (37). The use of chloroquine and hydroxychloroquine for patients referred to hospitals with COVID-19 was previously acknowledged by the US Food and Drug Administration (38). According to current findings, Hydroxychloroquine has no direct effect on SARS-CoV-2 (39). Pre-exposure prophylaxis with Hydroxychroloquine did not diminish the incidence of COVID-19 (40, 41). WHO too does not recommend Hydroxychroroquine treatment for the prevention of COVID-19 since the drug has no effect on avoiding sickness, hospitalization, or mortality from COVID-19. When compared to the standard of care, data from the Solidarity study showed that hydroxychloroquine did not reduce mortality in hospitalized COVID-19 patients (42). Whereas various studies found that Ivermectin was effective when used as a prophylactic treatment to lower COVID-19 infection (43). However, “Therapeutics and COVID-19: living guideline” by WHO does not recommend the use of ivermectin in patients with COVID-19 except in clinical trials (44). The COVID-19 Treatment Guidelines Panel recommends against the use of any oral drugs including ivermectin and hydroxychloroquine for SARS-CoV-2 pre-exposure prophylaxis, except in a clinical trial (45). Even though, the Ministry of AYUSH suggested various measures (46–48) to improve immunity there is no scientific evidence available in this regard.

However, this study only looked at health workers who were unimmunized; hence it did not look into vaccination as a factor. The study was initiated in the initial stages of the pandemic before the vaccine was developed. Since the study continued to enroll the participants after the infusion of vaccination campaign, the study was restricted to study participants who have not taken the vaccination. Another potential limitation of the study would have been the Recall bias as the information was gathered regarding the previous 14 days from the day of the interview. Also, the information about the study participant’s IPC behaviors was self-reported, which might have led to overestimation. For confirmation, the IPC and hand hygiene procedures should have been observed directly; however, due to the restricted entry policies that were prevailing in most hospitals, this was not possible. Since, this study was conducted at a time when WHO did not recognize the aerosol transmission of COVID-19, the study did not capture the aerosol transmission of the disease resulting in poor risk assessment of workers. This would have resulted in an underestimation of risk. Hence, due to the diverse modes of transmission of infection, the study was unable to capture the true risk assessment. Another limitation of the study was that it only captured the adequacy of IPC training and adherence to IPC. However, the study did not capture the weaknesses and lacunae in the PCI protocol which was beyond the scope of the present study. However, analyzing the shortcomings of the existing PCI protocols and rectifying them is crucial for bringing about a substantial impact on IPC practices.

Therefore, this study was able to identify potential risk factors for contracting COVID-19 infection among health workers in the hospital setting. These factors need to be taken into consideration while planning for pandemic preparedness in the future. The study was able to highlight the need for implementing IPC programs with regular theoretical and practical sessions for all health workers. Hence, there should be a separate hospital infection control department with staff specifically trained and updated in IPC practices. They should provide training and re-training of health workers and frequently monitor their IPC practices like the use of PPE and proper hand hygiene practices. The administrators should also ensure that all recruits are trained when they join for duty and refresher courses need to be provided regularly. It is also equally important that administrators need to ensure the adequate availability of PPE at healthcare facilities and also have in place a pandemic preparedness plan for the future. Since health workers with co-morbidities were found to be at a higher risk of getting infected with COVID-19, appropriate hospital policies should be in place to address the occupational hazards faced by health workers alongside appropriate IPC policies. Therefore, there is an urgent need to implement uniform IPC policies and also to have a pandemic preparedness plan across all hospitals in India.

## Data availability statement

The raw data supporting the conclusions of this article will be made available by the authors, without undue reservation.

## Ethics statement

The studies involving human participants were reviewed and approved by Institutional ethics committee of Amrita Institute of Medical Sciences (IEC-AIMS-2020-COMM-133). The patients/participants provided their written informed consent to participate in this study.

## Author contributions

LG, CT, CR, UN, PBh, JK, RR, PP, AB, PBa, MM, AC, AS, MY, BA, MAh, and AR substantially contributed to the conception or design of the work, the acquisition, analysis, interpretation of data for the work, drafting the work or revising it critically for important intellectual content, final approval of the version to be published, and agreement to be accountable for all aspects of the work in ensuring that questions related to the accuracy or integrity of any part of the work are appropriately investigated and resolved. All authors contributed to the article and approved the submitted version.

## Funding

This work received financial support from WHO for the conduct of the study as part of WHO multicenter case control study on “Risk factors of SARS-CoV-2 infection among health care workers in India.” But has not received any financial support for publication.

## Conflict of interest

The authors declare that the research was conducted in the absence of any commercial or financial relationships that could be construed as a potential conflict of interest.

## Publisher’s note

All claims expressed in this article are solely those of the authors and do not necessarily represent those of their affiliated organizations, or those of the publisher, the editors and the reviewers. Any product that may be evaluated in this article, or claim that may be made by its manufacturer, is not guaranteed or endorsed by the publisher.
